# Addressing Factors that Impact Sexual Well-Being and Intimacy in IBD Patients

**DOI:** 10.1007/s11894-024-00956-2

**Published:** 2025-01-09

**Authors:** Samantha Elias, Neilanjan Nandi, Simona Fourie, Lorraine Grover, Kira L. Newman

**Affiliations:** 1 Division of Gastroenterology and Hepatology, University of Pennsylvania, Philadelphia, PA USA; 2 Radcliffe Department of Medicine, University of Oxford, Oxford, UK; 3 The London Clinic, London, UK; 4 Department of Medicine, University of Michigan, Ann Arbor, MI USA

**Keywords:** Sexual health, Intimacy, Dyspareunia, Erectile dysfunction, Sexual and gender minority, Anoreceptive intercourse, Ostomy, Pelvic floor dysfunction

## Abstract

**Purpose of Review:**

This review details the pathophysiologic mechanisms from medical, surgical to psychosocial factors that illustrate how and why sexual health and intimacy are impacted in IBD.

**Recent Findings:**

Recent clinical surveys of practicing gastroenterologists document that clinicians should routinely address sexual health when addressing patient reported outcomes but very few actually make direct inquiry or suggest management into this important aspect of human life. Example ‘patter’ are suggested to clinicians to demonstrate how to introduce the subject of sexual intimacy and well-being and engender patient trust on this sensitive topic. Once specific symptomatology are elicited, then a review follows on how referral to a cadre of available multidisciplinary specialists can help directly manage the patient’s concerns. Specific emphasis on addressing the sexual health in ostomate and sexual and gender minority populations is focused upon as well.

**Summary:**

Overall, this in depth review highlights a practical clinical approach to understanding how to address sexual wellbeing and human intimacy in IBD patients.

## Overview

### Prevalence & Pathophysiology of Sexual Dysfunction in IBD

Sexual dysfunction in IBD is common and may be under reported. The rate of sexual dysfunction in IBD patients ranges from 40–60% in females and 40–50% in males [1]. It is often associated with prior operation, concurrent depression, high disease activity, and the use of steroids [2]. Another population similarly impacted by sexual dysfunction are pelvic cancer patients, who report similar symptoms of depression and decreased sexual function related to chemotherapy, radiation, and surgical treatments [3]. A recent meta-analysis demonstrated a statistically significant association between IBD and low sexual function scores. The presence of IBD was associated with increased risk of erectile dysfunction, low sexual satisfaction, poor performance on orgasm, poor overall desire, satisfaction, and quality of sexual function (95% CI: − 0.33 to − 0.15, *P* < 0.0001) [4].

The pathophysiology of sexual dysfunction is multifactorial and involves a complex process of desire, arousal, orgasm, and resolution. Desire is defined as an interest in sexual activity that hinges on initiation and receptiveness to sexual activity that can be dependent on an individual’s sense of body image, relationship status, and other psychosocial factors. IBD patients often experience poor body image related to prior surgeries or ostomy, issues with partner communication, and are at higher risk for depression increasing their risk for low desire at baseline. Arousal is defined as a physiologic response to sexual activity and may include increased blood flow to genitals, vaginal lubrications, clitoral swelling, and penile erection. Patients with IBD can experience interruption of these functions due to nerve damage from surgery or perianal disease or from medications like corticosteroids, making arousal difficult to achieve. Females may experience decreased vaginal lubrication with dyspareunia and males may experience inability to achieve erection or painful retrograde ejaculation. Orgasm is defined as an intense period of pleasure that involves involuntary muscle contractions and release of tension. These functions can be dysregulated in IBD patients from surgery or inflammation, leading to an inability to reach orgasm. The final step is resolution, which is defined as normalization following sexual activity that results in a physical and emotional well-being and fatigue. This sensation can often be short-lived or dampened in IBD patients with ongoing pain, inflammation, or prior nerve damage.

### Conversations About Sexual Dysfunction in IBD

Improving sexual well-being in IBD patients begins with creating a space to address these concerns in clinical encounters. These conversations involve sensitive topics. Therefore it is prudent to assess when and how to broach these conversations. Ideally a gastroenterologist would develop a strong rapport with the patient beforehand to help create a comfortable, open environment to facilitate sharing and dialogue. When to bring up the subject of sexual well-being with patients is difficulty as clinic visits can be very busy, especially during times of active disease management. While patients with active disease are likely encountering issues with sexual dysfunction, it may be more appropriate to defer the subject until disease is less active. A “well visit” is likely a prime opportunity to incorporate a discussion about sexual health history, sexual partners, current practices, and sexual function.

When bringing up the topic of sexual well-being, it is critical to normalize the topic and emphasize that the questions asked are routinely inquired in all IBD patients. Some examples of suggested phrases and wording include:

- *“Just so you know, I ask these questions to all of my adult patients, regardless of age, gender, or marital status. These questions are as important as the questions about other areas of your physical and mental health to provide personalized care.”*
- *“I am going to ask you a few questions about your sexual health and sexual well-being. I understand that these questions are very personal, but they are important for your overall health.”*
- *“Like the rest of our visits, this information is kept in strict confidence.”*
- *“Do you have any questions before we get started? “*

Once the conversation has begun, be equipped with an agenda of questions to ask along with resources to provide the patient once possible issues arise. The art of the ‘pause’ is critical to note. Pausing to permit time for patients to gather themselves and respond rather than filling silence with the next clinician-initiated dialogue promotes a safe space to share. The remainder of this paper will delve into tools for assessing sexual dysfunction, risk factors to consider when asking patients about their sexual well-being, and recommended treatment approaches to help gastroenterologists craft an agenda and create an individualized plan to help improve the sexual well-being of their IBD patients.

### Validated Tools for Assessing Sexual Dysfunction

Various assessment tools and validated scores that can be used for the assessment of sexual dysfunction. Some of the more commonly used tools are included below. Of note, some surveys are long thus difficult to administer during office visits due to time constraints; many of these assessments have not been validated for use in sexual and gender minority populations; and administering these surveys lacks purpose if no interventions are put in place to follow up the score.

**Table:** 

Male			
Questionnaire	Number of items	Factors assessed	Intended use
IBD-Specific Male Sexual Function Scale (IBD-MSDS)	10	(1) desire (2) initiation (3) guilt/fear (4) pain, lack of energy (5) bowel movements, bleeding, discharge	IBD-specific scale for clinical practice settings [5]
International Index of Erectile Function (IIEF)	5	(1) erectile function (2) orgasmic function (3) sexual desire (4) intercourse satisfaction (5) overall sexual satisfaction	Non-IBD-specific scale for research and clinical settings [6]
Sexual Health Inventory for Men (SHIM)	5	(1) erectile function (2) severity of erectile dysfunction	Created as an abridged version of IIEF to be administered in clinical settings and used for clinical assessments or research [7]
Brief Sexual Function Inventory (BSFI)	11	(1) sexual drive (2) erection (3) ejaculation (4) perceptions of problems in each area (5) overall sexual satisfaction	Assessment of male sexual function in clinical setting primarily used in urology clinics [8]
Female			
Questionnaire	Number of items	Factors assessed	Intended use
IBD-Specific Female Sexual Dysfunction Scale (IBD-FSDS)	10	(1) desire (2) initiation (3) guilt/fear (4) pain, lack of energy (5) bowel movements, bleeding, discharge (6) relationship initiation	IBD-specific scale for clinical practice settings [9]
Female Sexual Function Index (FSFI)	19	(1) desire (2) arousal (3) lubrication (4) orgasm (5) satisfaction (6) pain	Non-IBD-specific scale for research and clinical settings. [10]
Brief Index of Sexual Functioning in Women (BISF-W)	22	(1) thoughts/desires (2) arousal (3) frequency of sexual activity (4) receptivity/initiation (5) pleasure/orgasm (6) relationship satisfaction (7) problems affecting sexual function	Non-IBD-specific scale for research and clinical settings [11]
Sexual Function Questionnaire (SFQ)	32	(1) desire (2) physical arousal-sensation (3) physical arousal-lubrication (4) enjoyment (5) orgasm (6) pain (7) partner relationship	Developed to evaluate efficacy of drugs to treat female sexual dysfunction in clinical trials [12]

### Risk Factors for Development of Sexual Dysfunction in IBD

Often multiple, overlapping risk factors contribute to the development of sexual dysfunction in IBD patients. Inquiring about each of the below factors can identify which are contributing to a patient’s sexual dysfunction. Suggested considerations and management approaches are also listed below. We hope this aids in creating an individualized plan for each patient to help improve their symptoms.

#### Disease Activity

Active disease is associated with symptoms of sexual dysfunction. Studies demonstrate that both males and females with active IBD had decreased scores on sexual dysfunction scales than patients in remission or healthy controls. Specifically, male patients with active disease reported reduced orgasm, desire, and sexual satisfaction compared to patients in remission. Female patients with active disease reported decreased lubrication and increased pain compared to patients in remission [13]. In terms of phenotypic disease type, active perianal disease is most likely to lead to increased sexual dysfunction, especially in females [OR = 13.05 (2.32–73.44)] [14]. An important extraintestinal manifestation of IBD to be aware of is vulvovaginal Crohn’s disease, as this is an often overlooked area of disease presentation that can significantly impact patients sexual well-being and relationships [15].

#### Medications

Some patients report medications used to control their IBD having a negative impact on their sexual function. Long-term use of prednisone has been reported to cause a decrease in testosterone leading to decreased libido and erectile dysfunction [16] and increased rates of recurrent vulvovaginal candidiasis [17] while biologic medications commonly used in IBD have not been shown to have impact on sexual function [18]. Currently not enough data are available on small molecule impact on sexual function. Depression is a common co-morbidity in IBD and many patients are started on selective serotonin reuptake inhibitors (SSRIs). It is important to remember that SSRIs can lead to erectile dysfunction, decreased vaginal lubrication, and impair orgasm. Antidepressants with less risk of sexual side effects include serotonin and norepinephrine reuptake inhibitors (SNRIs), mirtazapine, and bupropion [19]. Chronic use of opioid medications, while increasingly avoided in IBD patients, can also lead to sexual dysfunction through the decreased release of luteinizing hormone leading to less production of testosterone and estradiol causing decreased libido and increased erectile dysfunction [20].

#### Surgical Consequences and Complications

Sexual dysfunction after surgery is a risk in IBD patients due to possible muscle and nerve damage to the pelvic floor. Patients reported their most common concerns involve loss of intimacy and sexual drive, ability to perform sexually, and overall decreased body image [21]. Most of the studies looking into these concerns focus on the most common IBD surgeries, ileal pouch anal anastomosis (IPAA). In a survey of sexual function post-IPAA, patients reported low occurrence of severe sexual dysfunction and overall improved quality of life [22]. Another study found patients that underwent successful IPAA experienced no adverse effects on sexual function and twelve months post-surgery both sexes reported improved quality of life and sexual functioning [23]. However, other studies have shown up to a 10% chance of developing erectile dysfunction after IPAA, although greater than 80% of patients report improvement of their erectile dysfunction after initiating medication treatment [24].This highlights the importance of asking about sexual functioning post-surgery to make sure these patients are identified and treated.

#### Psychosocial Factors

A myriad of psychosocial factors can contribute to sexual dysfunction in IBD, but there are a few important points to emphasize. Co-morbid depression and anxiety are extremely common in IBD. Multiple studies have identified depression as one of the most important determinants of sexual dysfunction in IBD patients and a key risk factor for poor sexual well-being in this patient population [25]. The mechanism of disease in IBD can lead to weight loss/gain, medication side effects, pain, decreased functioning of sexual organs, and/or surgical interventions with possible stoma creation. Qualitative studies found patients struggle with altered body image and sociocultural stigma against stomas and which negatively impacts their sexual well-being [26]. These factors understandably lead to a decrease in self-esteem and body image, decreased sexual desire and self-pleasure, poor communication in existing sexual relationships, or lack of confidence in pursing new relationships.

#### Tobacco Use

Cigarette smoking is a well-known cause of sexual dysfunction. Evidence shows a dose-dependent association between tobacco use and sexual dysfunction including male erectile dysfunction, female genital pain, female difficulty with arousal and reaching orgasm, and decreased libido in both sexes [27]. No studies specifically address the impact of tobacco on sexual functioning in IBD patients, however tobacco use is thought to cause disruption of endothelial functioning leading to poor blood flow and eventual endothelial damage [28]. IBD patients are already at high risk of tissue damage and poor blood flow from their disease state, so advising them to abstain from tobacco use is important for their sexual well-being.

### Non-IBD Specific Sexual Dysfunction Issues and Recommended Management

#### Ostomy/Stoma Management

Patients with a current or prior ostomy/stoma are at increased risk for poor body image and sexual dysfunction [29]. Similar health-related outcomes are observed in patients who undergo IPAA vs ileostomy creation, however patients who underwent IPAA had better scores in sexuality and body image that those with ostomies [30]. A systematic review found sexual dysfunction in patients with ostomies was attributable to a combination of psychologic factors (low self-esteem, low body image), physical factors (type of surgical resection, recovery time, complications), and the reaction to these changes by a patient’s sexual partner [31]. Unfortunately these factors are not just present in the post-op period, but can remain present for years afterwards. This underscores the need to continually re-assess the sexual well-being of patients with ostomies even years after surgery and disease remission. Some general recommendations for patient struggling with sexual activity while living with an ostomy are: empty their pouch before sexual intercourse, consider purchasing ostomy camouflage products like female lingerie and male high-waisted underwear, and use of oral and pouch deodorizer products if needed.

#### Erectile Dysfunction

Erectile dysfunction (ED) is a common issue encountered by male IBD patients. A study investigating post-proctectomy ED after rectal excision in cancer patients found that sildenafil completely reversed or satisfactorily improved ED in 79% of patients, and therefore should be highly considered in post-surgical IBD patients experiencing ED [32]. First-line intervention for ED is initiation of PDE5 medication (i.e. sildenafil, tadalafil, etc.). Patients should be instructed to take this medication four hours prior to sexual intercourse and be counseled on side effects of hypotension and priapism. Other options to treat ED warrant referral to urology and include topical alprostadil (Vitaros), intraurethral alprostasil (MUSE), rubber band or erection ring alongside a vacuum constrictive device, penile implants (malleable, inflatable), and intra-cavernosal injections. Of note, intra-cavernosal injections are generally most feared due to their invasive nature but have high efficacy rates [33]. Beyond pharmacologic and surgical intervention, there are also sexual health focused urologists and psychosocial therapists who specialize in ED.

#### Atrophic Vaginitis

Atrophic vaginitis is defined as thinning and drying of the vaginal walls with decreased vaginal secretions due to decrease in estrogen production. Symptoms include vaginal dryness and pruritis. Treatments include lubricants, topical or intravaginal estrogen therapy, or estrogen replacement therapy (transdermal or oral). Of note, estrogen replacement therapy should be considered carefully in patients with higher risk of blood clots such as patients with severe IBD, recent surgery, or a prior history of blood clots. Patients should avoid irritating lubricants with parabens, glycerin, or propylene glycol. They should also avoid petroleum and oil-based lubricants as they leave a coating in the vagina/rectum that traps bacteria.

#### Dyspareunia

Dyspareunia is defined as painful intercourse. Symptoms include pain with penetration, during deep thrusting, and/or throbbing lasting hours after sexual intercourse. First and foremost, patients should be treated for active disease and examined to assess for vulvar Crohn’s Disease as dyspareunia can manifest this way. Patients should also be asked about any symptoms of vaginal candidiasis and treated for symptoms if present. If pain is ongoing after these steps, other suggestions include planned toileting or emptying their ostomy pouch prior to sexual intercourse. Additionally, patients can consider use of silicone rings to reduce pain with deep penetration. All patients with dyspareunia should also be considered for referral to pelvic floor physical therapy, during which they can also discuss vaginal dilators and shown how to use them correctly.

#### Pelvic Floor Dysfunction

Pelvic floor dysfunction is a constellation of symptoms related to the inability to correctly relax or contract coordinated pelvic floor musculature. Symptoms include a variety of complaints, but most commonly involve urinary incontinence, constipation, fecal incontinence, pain or reduced sensation with intercourse. Referral to pelvic floor physical therapy can help patients develop a stronger connection between their brain and pelvic floor muscles and learn exercises to aid in controlling their symptoms.

#### Fecal Incontinence

Fecal incontinence is common in IBD and an important symptom to ask about. Studies have found that when asked, around 74% of IBD patients report at least occasional fecal incontinence. Most patients report fecal incontinence related to disease activity, although some patients have reported continued symptoms even during disease remission [34]. Fecal incontinence can be a result of overactive bowel, increased rectal sensitivity, damage to muscles or nerves from inflammation or prior surgery, or perianal fistulas. Management of fecal incontinence includes referral to pelvic floor physical therapy and biofeedback. In addition, patients can try planned bowel emptying prior to sexual intercourse to decrease risk of fecal incontinence during sexual activity which can be particularly helpful in patients who underwent IPAA.

#### Urinary Incontinence

Patients with IBD can also experience urinary incontinence that effects their sexual activity. *Stress urinary incontinence (SUI)* is defined as involuntary urinary leakage due to increased intra-abdominal pressure associated with coughing, sneezing, or laughing. It classically occurs with routine daytime activity and physical activity but can also occur during vaginal penetration. Non-surgical treatments include pelvic floor therapy, incontinence pessary or incontinence tampons. Surgical treatments include urethral bulking agents, native tissue repair, synthetic bladder slings, or artificial urinary sphincter, though candidacy for surgery may depend on IBD activity, history, and anatomic areas affected. *Overactive bladder (OAB)* involves symptoms of urinary urgency and incontinence with predominately nocturnal symptoms. Treatments include behavioral modifications (fluid management, bladder training, pelvic floor therapy), medications (beta-3-agonists, antimuscarinics, botox), or procedural (sacral neuromodulation, posterior tibial nerve stimulation).

### Sexual and Gender Minority Related Concerns and Management

Data about sexual health and sexual dysfunction in sexual and gender minority people with IBD are lacking. Sexual and gender minorities include people who identify as lesbian, gay, bisexual, asexual, transgender, nonbinary, Two Spirit, queer, intersex, or as having other non-heterosexual or non-cisgender sexual or gender identities [35]. At least 7.1% of general US population identifies as a sexual and gender minority and 20% of generation Z (birth: 1997–2012) identify as a sexual and gender minority [36]. Sexual and gender minorities have diverse sexual practices that overlap with those of heterosexual and cisgender individuals, but there are some considerations that may be more pertinent for sexual and gender minority people with IBD. It is imperative that gastroenterologists approach patients with an open and non-judgmental attitude and not make assumptions about their patient’s sexual or gender identity or sexual practices to allow for therapeutic alliance and clear communication about the patient’s experiences and needs.

#### Anoreceptive Intercourse

For some sexual and gender minorities, the ability to partake in pleasurable anoreceptive intercourse is crucial to sexual well-being. However, there is limited evidence-based guidelines for IBD patients engaging in anoreceptive intercourse. Local inflammation from active IBD can cause anal fissures, skin tags, proctitis, strictures, and fistula formation thus patients with active perianal disease are generally instructed to avoid anoreceptive intercourse as physical manipulation of the perianal region can exacerbate inflammation [37]. However, no data exists on when it's safe to resume anoreceptive intercourse and can be especially limiting for patients with difficult to control perianal disease. Furthermore, some of the most common surgeries for IBD involve altering rectal anatomy. In IPAA, the rectum is removed and the ileum is attached to the anus which results in decreased elasticity and increased risk of suture damage and rupture of the ileal wall with anoreceptive insertion. In ileorectal anastomotic surgery, the rectum is preserved which makes anorecptive intercourse more feasible, however comes with a risk of developing pouchitis resulting in more painful intercourse [38]. In proctocolectomy with end ileostomy, the entire colon and rectum are removed which in showed no statistically significant impact on male and female sexual function, although reports of post-surgical erectile dysfunction and increased pain with vaginal penetration were reported [39]. Of note, for patients with perianal fistulas knotless seton placement is associated with less pain than knotted setons which leads to more pleasurable anoreceptive intercourse [40]. Overall, open discussions with patients about their sexual practices alongside clear communication about their active perianal disease symptoms and surgical procedures with is paramount to optimizing sexual well-being.

#### Gender Affirming Care

Another important consideration for sexual and gender minorities with IBD is gender-affirming care. No specific data exists exploring the sexual well-being of transgender individuals with IBD, but meta-analyses have investigated how to better support sexual well-being in transgender individuals overall. Studies have explored sexual desire, arousal, orgasm, pain, and sexual satisfaction in both transmen and transwomen. Overall, sexual satisfaction improved with testosterone, estrogen and antiandrogen therapy, and/or gender-affirming surgery [41]. One study demonstrated no increase in flare after starting gender affirming hormone (GAH) therapy unless there was active IBD identified prior to GAH initiation (univariate 58% vs 24%, *p* = 0.003; multivariate OR 5.1 [95% CI 1.7–15.2]). Trans men were more likely to experience flare after GAH initiation than transp-women (trans-men: 51% vs trans-women: 26%; *p* = 0.02) [42]. Undergoing gender-affirming surgery in IBD patients must be carefully considered as preexisting perianal and rectal inflammation can impact tissue quality that is needed for successful pelvic reconstruction [43]. In individuals who have postoperative pain or sexual dysfunction following gender-affirming surgeries, clinicians should refer patients for pelvic floor rehabilitation.

#### HPV Screening

Another question that often arises is the utility of anal cytology and/or anal human papilloma virus (HPV) screening in IBD patients on immunosuppressive medications who engaged in anoreceptive intercourse. These patients are in theory at higher risk for HPV infection and progression to anal cancer. Per the International Anal Neoplasia Society (IANS), certain patients with higher risk of developing anal cancer are patients living with HIV, men who have sex with men, women with HPV-related precancerous lesions or cancer, solid organ transplant recipients, and patients with autoimmune diseases [44]. The IANS recommends screening for anal cancer in men who have sex with men (MSM) with HIV and transgender women (TW) with HIV starting at age 35, men who have sex with women (MSW) and women with HIV at age 45, MSM and TW without HIV at age 45, history of vulvar high-grade squamous intraepithelial lesion (HSIL) or cancer within 1 year of diagnosis, solid organ transplant recipients ten years post-transplant. They recommended shared decision making about anal cancer screening in patients with autoimmune conditions like IBD at age 45 [45]. Patients with IBD who are receiving immunosuppression are at a 30% increased risk for cervical neoplasia compared to the general population as persistent HPV infection and a suppressed immune system can predispose to cervical and anal cancer [46]. The CDC currently recommends the HPV 9-valent vaccine (Gardasil 9) for all patients between the ages of 9 and 26 years old and shared clinical decision making about receiving the vaccine for patients between the ages of 27 to 45 years based on their risk for acquisition of HPV [47].

### Impact of Sexual Dysfunction on Quality of Life and Relationships

Sexuality is integral to a person’s personality as it drives their feelings, thoughts, and behaviors towards others. A large U.S. population study published in 2017 found participants rated sexual health and satisfaction as highly important to their quality of life. Sexual well-being is therefore a fundamental part of quality of life and overall health. However, many health-related measures often do not incorporate sexuality as its own quality of life measurement. Most surveys include a section assessing a relationship, but do not specifically address sexual well-being as a distinct category. Participants with poor health reported low sexual satisfaction and indicated sexual satisfaction an important factor in their quality of life [62.2% of men (95% CI = 59.4–65.0) and 42.8% of women (95% CI = 39.6–46.1, *P* < 0.001)] [48].This supports the need for routine clinical assessment of sexual well-being in IBD patients.

Active enterocolonic IBD can inherently impact adjacent sexual organs. A 2020 study assessing sexual quality of life in IBD patients, documented higher levels of frustration, anxiety, depression, embarrassment, and loss of self-confidence when compared to controls. 17% of IBD females reported not feeling well in their own skin and 42% reported attempting to avoid sexual activity all together. Many voiced concerned related to emotional, relationship, and self-esteem issues [49].This emphasizes the need to aggressively address symptoms of anxiety and depression, relationships, and social isolation in patients in IBD patients.

Clinicians must encourage patients to have open and clear communication with their partner, as lack thereof can lead to feelings of tension and guilt on both sides while good communication can relieve anxiety and help improve sexual satisfaction. Delaying these conversations can delay building trust that is needed for satisfying sexual encounters and may promote voluntary childlessness [50]. There is also a vast difference between patients who have been in long-term relationships compared to those trying to navigate starting a new relationship and how to disclose their disease and communicate effectively about their sexual needs. Overall, the initiative should be taken by the gastroenterologist to broach questions about well-being as research shows sexual well-being is an important marker of quality of life. However, fully addressing patient concerns should involve a multidisciplinary with referral to psychologists and other specialists as needed to support IBD patients’ relationships and partner communication as this will ultimately lead to better long-term support and solutions that cannot be provided in the gastroenterology office alone.

## Conclusions and Future Directions

Multiple modifiable risk factors influence sexual well-being and intimacy in IBD patients. Addressing sexual and gender minority needs proactively is necessary. Clinicians should remove the burden from the patient and take initiative to broach the subject of sexual health themselves. It is important to keep in mind body image dissatisfaction is a common in post-surgical, ostomate, and IBD populations. Co-morbid anxiety and depression play a central role in the patient experience. Gastroenterologists should not be expected to manage sexual dysfunction in their patients alone. Triage to appropriate subspecialists and utilizing multidisciplinary team members is integral to success as each offers their own expertise and leads to better patient outcomes.

### Key Takeaways

**Table:** 

Ask	*Ask the questions* about intimacy concerns, body image concerns, erectile dysfunction, psychosocial well-being (depression/anxiety), symptoms of pelvic floor dysfunction
Assess	*Assess* disease activity, indices/surveys, sexual activity indices and questionnaires, medication side effects, pre and post operative sexual health,
Educate	*Educate* about tobacco cessation, importance of optimal medical therapy, ostomy websites/pamphlets, IBD support group, adherence to IBD medications
Refer	*Refer* to PCP, mental health provider, subspeciality providers (gyn, urology, colorectal surgery), ostomy nurse experts

## Key references

- Zhang, J., Wei, S., Zeng, Q. et al*.* Prevalence and risk factors of sexual dysfunction in patients with inflammatory bowel disease: systematic review and meta-analysis. *Int J Colorectal Dis* **36**, 2027–2038 (2021). 10.1007/s00384-021-03958-y
  - ○ Excellent meta-analysis and overview of the prevalence of sexual dysfunction in patients with inflammatory bowel disease. This reference provides specific data analysis that highlights how common sexual dysfunction is in both male and female IBD patients and therefore why it is an important topic for research and discussion.
- Knowles SR, Gass C, Macrae F. Illness perceptions in IBD influence psychological status, sexual health and satisfaction, body image and relational functioning: A preliminary exploration using Structural Equation Modeling. *J Crohns Colitis* 2013;7:e344-50. 10.1016/j.crohns.2013.01.018
  - ○ Comprehensive overview of how IBD disease activity impacts patients self-perceptions, mood, interpersonal relationships, and sexual functioning and desire. This research study provides an organized view of the complex impact IBD as an illness has on patients’ perceptions and social function that are important to their sexual and relationship satisfaction.
- Fourie S, Jackson D, Czuber-Dochan W, Norton C. A Decade of Waiting: Experiences of Women Living With Vulvar Crohn's Disease and Interactions With Healthcare Professionals Related to Their Sexual Well-Being: A Qualitative Study. Crohns Colitis 360. 2023 May 12;5(3):otad025. 10.1093/crocol/otad025 PMID: 37,250,190; PMCID: PMC10212277.
  - ○ Illuminating qualitative study on the interactions of patients with vulvar Crohn’s Disease and their interactions with health care workers leading to delayed diagnosis. While vulvar Crohn’s Disease is rare, the qualitative data gathered in this article provides an overview of themes important to sexuality and its connection to quality of life that are important to address in patients living with IBD.
- Roseira, J, Magro, F, Fernandes, S, Simões, C, Portela, F, Vieira, AI, Patita, M, Leal, C, Lago, P, Caldeira, P, Gago, T, Currais, P, Dias, CC, Santiago, M, Dias, S, Tavares de Sousa, H. Sexual Quality of Life in Inflammatory Bowel Disease: A Multicenter, National-Level Study, *Inflammatory Bowel Diseases*, Volume 26, Issue 5, May 2020, Pages 746–755.10.1093/ibd/izz185
  - ○ Large, multi-center study conducted in Portugal that investigated sexual quality of life in patients with IBD. This comprehensive overview focuses not only on physical sexual dysfunction but also on social and emotional elements that contribute to negative sexual quality of life in patients with IBD. This study provides a great roadmap for assessing all aspects (physical, social, emotional) that impact sexual desire and function – and specifically the issues impacting the IBD population.

## Data Availability

No datasets were generated or analysed during the current study.
